# Advanced Hemodynamic Monitoring Allows Recognition of Early Response Patterns to Diuresis in Congestive Heart Failure Patients

**DOI:** 10.3390/jcm12010045

**Published:** 2022-12-21

**Authors:** Maya Dagan, Yotam Kolben, Nir Goldstein, Arik Ben Ishay, Meir Fons, Roei Merin, Arik Eisenkraft, Offer Amir, Rabea Asleh, Arie Ben-Yehuda, Dean Nachman

**Affiliations:** 1 Heart Institute, Hadassah Medical Center, The Hebrew University of Jerusalem, Jerusalem 9112102, Israel; 2 Department of Internal Medicine, Hadassah University Medical Center, Faculty of Medicine, Hebrew University of Jerusalem, Jerusalem 9112102, Israel; 3 Biobeat Technologies Ltd., Petah Tikva 4951126, Israel; 4 Institute for Research in Military Medicine, Faculty of Medicine, the Hebrew University of Jerusalem, the Israel Defense Force Medical Corps, Jerusalem 9112102, Israel; 5 Azrieli Faculty of Medicine, Bar-Ilan University, Zfat 1311502, Israel

**Keywords:** congestive heart failure, diuresis, remote patient monitoring, personalized medicine, digital health

## Abstract

There are no clear guidelines for diuretic administration in heart failure (HF), and reliable markers are needed to tailor treatment. Continuous monitoring of multiple advanced physiological parameters during diuresis may allow better differentiation of patients into subgroups according to their responses. In this study, 29 HF patients were monitored during outpatient intravenous diuresis, using a noninvasive wearable multi-parameter monitor. Analysis of changes in these parameters during the course of diuresis aimed to recognize subgroups with different response patterns. Parameters did not change significantly, however, subgroup analysis of the last quartile of treatment showed significant differences in cardiac output, cardiac index, stroke volume, pulse rate, and systemic vascular resistance according to gender, and in systolic blood pressure according to habitus. Changes in the last quartile could be differentiated using k-means, a technique of unsupervised machine learning. Moreover, patients’ responses could be best clustered into four groups. Analysis of baseline parameters showed that two of the clusters differed by baseline parameters, body mass index, and diabetes status. To conclude, we show that physiological changes during diuresis in HF patients can be categorized into subgroups sharing similar response trends, making noninvasive monitoring a potential key to personalized treatment in HF.

## 1. Introduction

Heart failure (HF) care is a pressing need with a prevalence of 1–2% in major industrialized countries and the significant load it exerts at the patient and society levels [1,2,3]. HF is characterized by a progressive course of disease accompanied by high hospital readmission rates, which account for a major portion of the economic burden [4]. Improving ambulatory management has been a crucial component in the efforts to reduce hospitalizations and improve mortality [5].

Diuretics remain a cornerstone in HF management, yet there are no clear guidelines on how to adjust their administration at home or in ambulatory care [6]. Guidelines recommend monitoring daily weight changes, despite a debatable efficacy [7,8]. Data from invasive monitoring of intracardiac pressures suggest hemodynamic changes begin days to weeks before the deterioration becomes clinically visible [1,9,10]. Thus, there is an unmet gap to find reliable markers that help to tailor diuretic treatment and prevent deterioration [11].

HF is a heterogeneous syndrome with different etiologies, pathophysiology, and hemodynamic characteristics. A personalized approach to guiding HF treatment may lead to improved outcomes. The introduction of wearable monitoring devices and big data analysis capabilities has the potential to provide patient-level insights into real-time and continuous physiological status in the ambulatory setting. Moreover, some of these devices can continuously monitor advanced hemodynamic parameters such as Cardiac Output (CO), Stroke Volume (SV), and Systemic Vascular Resistance (SVR), which have specific importance in HF care.

This study aimed to assess whether advanced hemodynamic noninvasive monitoring could be utilized to recognize patterns of response to diuresis in HF patients, receiving intravenous diuresis in a single medical center outpatient clinic.

## 2. Materials and Methods

### 2.1. Ethical Considerations

The researchers had no personally identifiable information about the individuals included in the analysis.

The study was approved by the Hadassah Ein-Kerem medical center institutional review board (approval number 0364-20-HMO; NCT04548024). Enrolled participants provided written informed consent. All mentioned methods were carried out following relevant guidelines and regulations.

### 2.2. Study Participants

Patients above 18 years of age, diagnosed with heart failure regardless of ejection fraction, arrived at the Hadassah Ein-Kerem medical center outpatient clinic to receive intravenous diuresis.

### 2.3. Study Design

HF patients arriving for intravenous diuresis treatment were recruited. Upon their consent, detailed medical history was documented, as well as a list of medications and a recent echocardiography report. Patients with Ejection Fraction (EF) of 50% and above were defined as having HFpEF. Patients with mildly reduced EF (41–49%) and reduced EF (40% and below) were defined as having heart failure with reduced ejection fraction (HFrEF) [1]. Before and after treatment they were asked to rate their dyspnea using a visual analog scale (VAS), by making a line on a 10 cm horizontal line ranging from none (“I can breathe as I normally do”) to severe (“I cannot breathe at all”). Weight was recorded using a weighing scale (Healthweigh™, Shekel, Beit-Keshet, Israel) before and after diuresis. Obesity was defined as a body mass index (BMI) equal to or above 30 (calculated as weight in kilograms divided by the square of the height in meters). The wrist monitor was attached before treatment initiation and was removed upon completion. Physiological measurements were recorded every 60 s during diuresis. Finally, during a telephone visit on day 30 post-enrollment, data regarding cardiac and non-cardiac hospitalizations was obtained.

### 2.4. The Photoplethysmography-Based Monitoring Device

**Figure 1:**
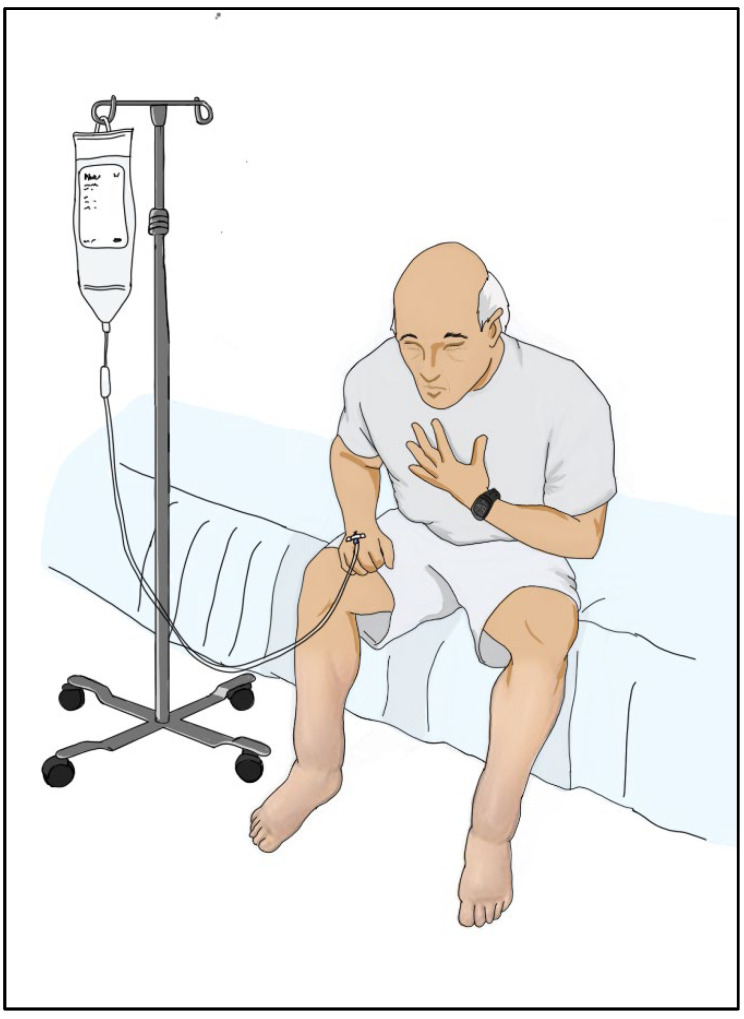
The photoplethysmography-based monitor in a wristwatch configuration. The device is shown to be worn by a heart failure patient while receiving intravenous diuretics.

The hemodynamic monitors (Figure 1) used in this study (BB-613WP, Biobeat Technologies Ltd., Petah Tikva, Israel) are based on reflective photoplethysmography (PPG) technology, in which part of the transmitted light is reflected from the tissues and detected by a photodiode detector positioned near the light source transmitter. The unique properties of the sensor allow it to capture slight changes in tissue reflectance, from which it derives measurements of several hemodynamic parameters, using Pulse Wave Transit Time (PWTT) combined with Pulse Wave Analysis (PWA) [12]. The device is applied to the wrist similar to a wristwatch and requires a single trimonthly calibration of blood pressure using a cuff-based device. The device monitors 12 parameters, including non-invasive cuffless systolic and diastolic blood pressure (SBP and DBP, respectively), pulse pressure (PP), mean arterial pressure (MAP), heart rate (HR), blood oxygen saturation (SpO_2_), respiratory rate (RR), SV, CO, cardiac index (CI), SVR, and body temperature. The device has CE mark approval for all parameters and FDA clearance for BP, HR, RR, body temperature, and SpO_2_.

### 2.5. Data Collection

Physiological data were collected by the PPG-based monitors and automatically transmitted to a cloud-based data storage, with no personal identifiers or any other protected health information (PHI) included. Unusual events were recorded in parallel by the research team. The devices did not provide real-time alerts, and the data were analyzed retrospectively.

### 2.6. Statistical Analysis

To compare patients receiving diuresis at unequal time intervals, we compared patients based on the percentage of treatment duration, and the change in parameters was assessed along with the quartiles of treatment duration. Mixed ANOVA was used to test for time-sex, weight, and HF-type interaction. Repeated measures ANOVA was used to compare treatment duration quartiles. Between-two-groups comparisons were done using an independent-samples t-test for numerical parameters following Levene’s test for equality of variances. Chi-square was used for categorical parameter comparison between independent samples. ANOVA was used to compare more than 2 independent groups. In cases where a significant difference was achieved, Bonferroni posthoc test was used to identify the different pairs. All tests were 2-tailed and significance was defined as *p* < 0.05. A *p*-value between 0.05 and 0.1 was defined as a trend. Data appears as mean ± standard deviation.

K-means clustering (Scikit-learn K-means) was used to divide the cohort based on changes in physiological parameters. The elbow method was used to find the appropriate number of clusters, as a balance between the number of clusters and the smallest within-cluster sum of squares (WCSS) value. PCA was done with Scikit-learn as well. Data were scaled using Scikit-learn Standard Scaler.

## 3. Results

30 patients were enrolled, of which one patient was excluded due to technical reasons not related to the monitoring. The mean (±SD) age was 76.2 ± 9.2 years. 17 participants (60%) were self-specified as males. Demographic details, past medical history, and baseline hemodynamic parameters are provided in Table 1. The lowest EF was 15%. The average ± SD of EF was 60.2% ± 3.43% among the HFpEF patients, and 31.4% ± 11.4% among the HFrEF patients. Most of the data collection was performed before the adoption of the recent ESC heart failure GDMT guidelines. Thus, four pharmacology groups of medications were used in one patient only, as SGLT2i was introduced only recently into the ESC heart failure guidelines. 5 patients with HFrEF were treated with 3 medications following the previous ESC heart failure guidelines. 6 patients were treated with 2 medications, and 3 were treated with only one medication. 3 patients with HFrEF had cardiac resynchronization therapy (CRT) and 3 patients with HFrEF had implantable cardioverter defibrillators (ICD). In total, 6455 measurements were collected during an average monitoring period of 183 ± 53.6 min. All patients received furosemide as the diuretic agent. The mean dose of furosemide was 207.6 ± 51.7 mg. 15 patients (46%) received additional intravenous therapies along with furosemide, mainly Aminophylline and Iron supplements. The mean duration of diuresis administration was 183.7 ± 53.7 min. There were no adverse events from using the device and no patients were lost to follow-up. The average change in VAS score during the diuresis was −0.46 cm ± 0.9 (*p* = 0.016). The average weight change during diuresis was −1.1 ± 1.7% (*p* < 0.001). During the 30-day follow-up, two patients were hospitalized. One patient was admitted due to sepsis secondary to bacterial peritonitis and eventually died, and the other patient was hospitalized due to acute-on-chronic renal failure and discharged after returning to baseline creatinine levels.

**Figure 2:**
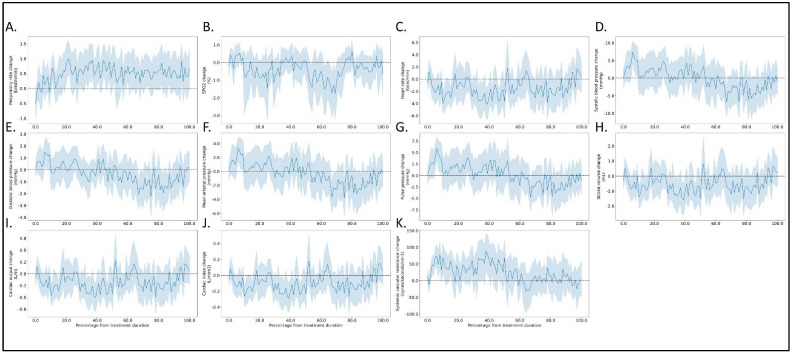
Relative changes from baseline in measured parameters during intravenous diuretic treatment. (**A**) respiratory rate (RR), (**B**) blood oxygen saturation (SPO_2_-%), (**C**) systemic vascular resistance (dynes/sec/cm^−5^), (**D**) heart rate (beats per minute), (**E**) stroke volume (milliliters), (**F**) cardiac output (liter/minute), (**G**) cardiac index (liter/minute/body surface area), (**H**) systolic blood pressure (mmHg), (**I**) diastolic blood pressure (mmHg), (**J**) pulse pressure (mmHg), (**K**) mean arterial pressure (mmHg).

**Figure 3:**
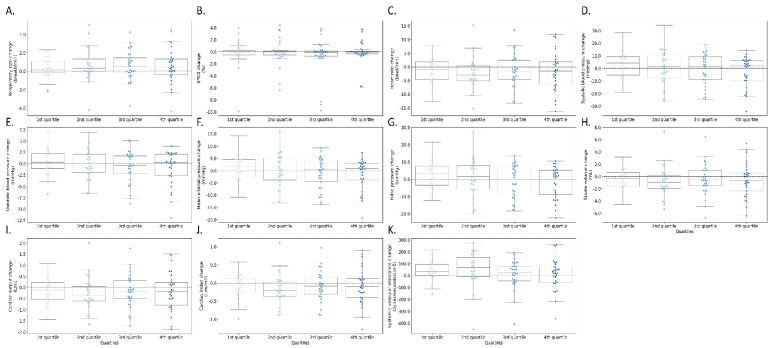
Relative changes from baseline in measured parameters along the quartiles of diuretic treatment. (**A**) respiratory rate (RR), (**B**) blood oxygen saturation (SpO_2_–%), (**C**) systemic vascular resistance (dynes/sec/cm^−5^), (**D**) heart rate (beats per minute), (**E**) stroke volume (milliliters), (**F**) cardiac output (liter/minute), (**G**) cardiac index (liter/minute/body surface area), (**H**) systolic blood pressure (mmHg), (**I**) diastolic blood pressure (mmHg), (**J**) pulse pressure (mmHg), (**K**) mean arterial pressure (mmHg).

Relative changes from baseline in measured hemodynamic parameters during the treatment phase and along the quartiles of treatment duration are presented in Figure 2 and Figure 3, respectively. There were no significant differences between any hemodynamic parameter values at initiation and the end of diuresis. Changes in those parameters were not significantly correlated with changes in the VAS dyspnea assessment and weight change.

**Figure 4:**
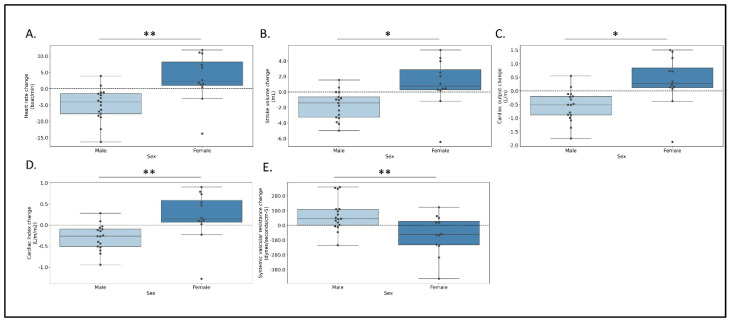
Significant relative changes from baseline in measured parameters from baseline to the fourth quartile of intravenous treatment stratified by sex. (**A**) heart rate (beats per minute), (**B**) stroke volume (milliliters), (**C**) systemic vascular resistance (dynes/sec/cm^−5^), (**D**) cardiac output (liter/minute), (**E**) cardiac index (liter/minute/body surface area). * *p* < 0.05, ** *p* < 0.01.

When stratifying the cohort based on sex, weight, and ejection fraction, we found sex-time interaction in several parameters including HR, SV, CO, CI, and SVR. When comparing the final stage of diuresis with baseline measurements (Figure 4), males had a reduction in CO (−0.6 ± 0.6 L/min) while in females CO increased (0.3 ± 0.9 L/min, *p* = 0.006). Similar findings were observed in SV (−1.7 ± 1.8 and 1.1 ± 3.1 mL, respectively, *p* = 0.011), CI (−0.3 ± 0.3 and 0.2 ± 0.6 L/min/m2, respectively, *p* = 0.017), and HR (−4.9 ± 5.0 and 3.2 ± 7.2 beats per minute, respectively, *p* = 0.003), while the SVR showed inverse association (67.0 ± 107.0 and −64.2 ± 134.9 dynes/sec/cm^−5^, respectively, *p* = 0.011). Significant differences were also found between patients with or without obesity when comparing baseline and last quartile measurements. SBP and PP did not change in the group without obesity, yet decreased in the group with obesity (SBP: −0.1 ± 12.0, −9.6 ± 10.0 mmHg; PP: 0.6 ± 8.4, −7.7 ± 7.7 mmHg; *p* = 0.046, *p* = 0.024, respectively). No significant differences were observed when stratifying the data based on ejection fraction.

**Figure 5:**
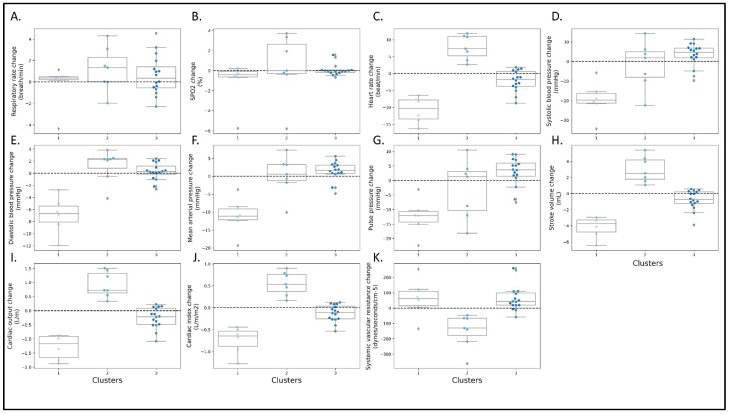
Relative changes from baseline in measured parameters from baseline to the fourth quartile of intravenous treatment in the three phenotypic clusters. (**A**) respiratory rate (RR), (**B**) blood oxygen saturation (SpO_2_-%), (**C**) systemic vascular resistance (dynes/sec/cm^−5^), (**D**) heart rate (beats per minute), (**E**) stroke volume (milliliters), (**F**) cardiac output (liter/minute), (**G**) cardiac index (liter/minute/body surface area), (**H**) systolic blood pressure (mmHg), (**I**) diastolic blood pressure (mmHg), (**J**) pulse pressure (mmHg), (**K**) mean arterial pressure (mmHg).

Finally, we used a k-means clustering algorithm to classify the patients into different groups based on their hemodynamic response patterns. Since some of the parameters are related or calculated from other parameters, we have decided to enter only changes in HR, SBP, DBP, CI, and SVR into the algorithm. Using the elbow method (see methods), we found that the ideal number of clusters should be 3 clusters (Figure 5). We found three different patterns of change that characterized each cluster. Patients in cluster 1 demonstrated a decrease in SBP, DBP, PP, MAP, SV, CI, CO, and increased SVR. Cluster 2 showed a somewhat opposite trend towards a rise in HR, DBP, SV, CO, CI, and a reduction in SVR. Cluster 3 demonstrated no immediate hemodynamic response (Figure 5).

**Figure 6:**
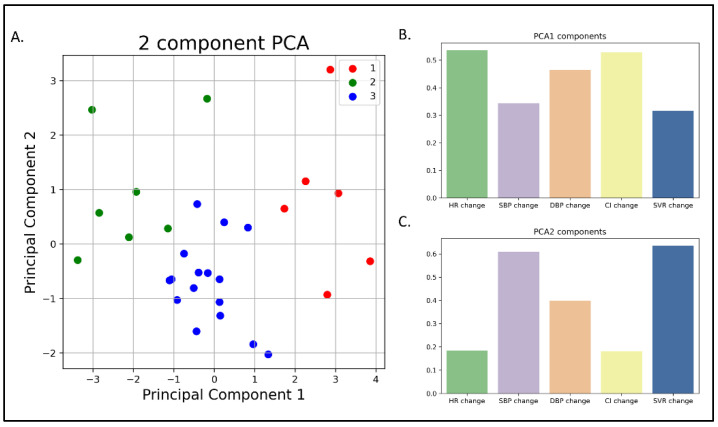
Principal Component Analysis (PCA). (**A**) Graphic representation of a principal component analysis (**A**) of the three phenotypic clusters. (**B**,**C**) The different parameters composing the components of the PCA.

Principal Component Analysis (PCA) was used for dimensionality reduction together with the k-means clusters (Figure 6). According to the PCA, the two principal components were responsible for 97% variation in the patients, where changes in HR, DBP, and CI contributed mainly to component 1, while SBP and SVR contributed mainly to component 2 (Figure 6).

We further looked at the baseline parameters/characteristics of patients in each cluster to understand if we could identify different characteristics that unify each cluster (Table 2). The main characteristic that appears to differ between clusters was the patients’ sex. Patients in cluster 1 were mainly males (5 out of 6), while mainly females were included in cluster 2 (6 out of 7). Therefore, we compared female patients in cluster 2, that exhibited elevation in several parameters at the end of the diuresis, with females in cluster 3, that showed no changes. We found that female patients in cluster 2 were significantly younger (71.3 ± 6.9 and 84.6 ± 4.0 years, respectively, *p* = 0.004), had higher baseline PP (70.3 ± 20.0 and 43.5 ± 15.6 mmHg, respectively, *p* = 0.037) and showed a trend towards lower CO (6.0 ± 0.5 and 6.8 ± 0.7 mL/min, *p* = 0.056) compared with female patients in cluster 3.

Although not significant, 5 out of 6 (83%) female patients in cluster 2 were diagnosed with pre/type 2 diabetes, while only one out of five (20%) in cluster 3 had a similar diagnosis. Additionally, all-female patients in cluster three had HF with preserved ejection fraction (HFpEF) type. Differences between male patients in cluster 1 (showed a reduction in several parameters) and cluster 3 (no apparent change) were not as clear as for female patients. Baseline PP and CI trended to be higher in male patients in cluster 1 than in cluster 3 (PP: 71.2 ± 12.1 and 49.8 ± 28.8 mmHg, respectively, *p* = 0.056; CI: 4.0 ± 0.4 and 3.6 ± 0.3 mL/min/m^2^, *p* = 0.051).

## 4. Discussion

In this study, we examined the hemodynamic changes during several hours of intravenous diuresis in HF patients. The literature describing the acute effects of diuresis on hemodynamic parameters is rather limited and archaic. Most studies found a reduction in CO and a rise in SVR after diuresis [13,14,15], but some describe the opposite [16,17]. This can be explained when considering that the pathophysiology underlying the effects of volume status on cardiac performance in HF is complex. While diuresis may affect preload and afterload, the intricacy of the neuro-hormonal response to diuresis cannot be simplified only by these two components. In this study, when looking at the entire cohort, we found that the hemodynamic parameters did not change significantly during diuresis, as opposed to previous studies that demonstrated some changes in advanced hemodynamic parameters. Additionally, we found no correlation between the clinical (weight and dyspnea) and the hemodynamic parameters, which can be attributed to the rather stable hemodynamic status and the non-acute setting of the diuresis sessions that led to minute, yet statistically significant clinical effects.

The effect of intravenous furosemide on BP is a source of debate. Most studies demonstrated no or minimal decrease in BP without any direct arterial vasoactivity [18,19]. Our findings strengthen these findings, as no net changes in BP were noted. It was suggested that some of the immediate effects of furosemide are driven by its venodilatatory effect, leading to reduced preload [20]. The used monitoring technology cannot monitor venous capacitance, and future studies with other technologies are needed to settle the debate.

Interestingly, when analyzing the hemodynamic changes from baseline to the last quartile of treatment, we found significant differences between males and females. Female participants had a lower relative change in SVR and higher in CO, CI, HR, and SV. We could not find previous data regarding the hemodynamic response to diuresis stratified by sex. Previous studies did find different outcomes in HF patients correlating with sex and different response to treatments [21,22]. While HFpEF is the most prevalent type of HF in women [23], it is important to note that in our study there was no significant association of response with HF type. Further research is needed to explore the mechanism underlying this link between sex and hemodynamic response to HF treatment and diuresis in particular.

As opposed to patients without obesity, patients with obesity had a reduction in BP at the end of diuresis. This phenomenon might correlate with previous data, showing that patients with obesity and with acute decompensated HF had a higher fluid loss volume after furosemide administration, compared with patients without obesity [24]. Yet, the mechanism is unclear.

As stated earlier, HF pathophysiology is rather heterogeneous and complex. Within the larger drive towards personalized medical care, the field of HF management can benefit from recognizing different phenotypes requiring different approaches. Thus, we attempted to find subgroups with different hemodynamic response patterns, using an unsupervised clustering algorithm. Finding such HF patterns could help in the future to tailor the most appropriate treatment protocol for each HF patient population, as well as improve the awareness of healthcare providers of specific HF patients that warrant closer supervision during treatment.

In the presented cohort, cluster 2 represents mainly females with a higher glomerular filtration rate (GFR). This cluster showed reduced SVR with improved CI at the end of diuresis, appropriate with the desirable changes according to the Frank-Sterling curve. Reduced Na-K-2CL cotransporter abundance and higher “furosemide saturation” in females [25], along with better furosemide delivery due to improved renal clearance, may explain the difference in response in this cluster. In contrast, cluster 1 is composed mainly of males with low GFR, with the lowest furosemide delivery and saturation. Cluster 3 is somewhat balanced, and all parameters are in the middle of the range between clusters 1 and 3. The influence of each parameter in a different direction, in this case, may explain the lack of mean cluster response. HbA1c levels were different among clusters, with the highest levels among cluster 2 participants. Multiple reports showed that diabetic patients with chronic HF and acute decompensated HF need higher doses of furosemide than non-diabetic patients [26,27]. Our findings may help better understand this phenomenon, demonstrating early hemodynamic response in this group.

The findings in this study were enabled using a wearable device that allows non-invasive monitoring of multiple cardiopulmonary parameters and analysis of multiple data points. New insights into the pathophysiology and hemodynamics of medical conditions will arise in the coming years, alongside the introduction of novel continuously advanced ambulatory monitoring capabilities. A larger cohort with a wider representation of the heterogenous patient population is also needed to better identify subgroups with different response phenotypes. Though the heterogeneity of the enrolled patients concerning baseline parameters such as HF type, any comorbidities, and medical therapy beyond the diuresis treatment is a limitation, this is a true representation of the real-life HF population, characterized by multiple comorbidities and polypharmacy. The participants also differed in the frequency of outpatient IV diuresis sessions, dosing of furosemide, and the coadministration of other medications. We did not find a significant correlation between hemodynamic parameters and the co-administration of other drugs alongside furosemide.

The clinical setting that we chose was ambulatory diuresis treatment among relatively stable patients, and they were not defined as decompensated. The aim was to define, among this patient population, the hemodynamic changes, that might allow us in the future, better adjustment of treatment, to prevent events of deterioration and reduced quality of life. Within this future effort, we will also include acute decompensated HF patients, follow the patients for a longer period, emphasize long-term clinical outcomes, look at the relationship between the use of diuretics and other medications and clinical endpoints, including readmission rates, quality of life, six-min. walk, etc. From the preliminary study presented here, and from the results of future studies, we intend to have a better understanding of the advanced personalized tailoring of treatment among this challenging population of patients.

## 5. Conclusions

To conclude, this study is the first step towards the implantation of advanced non-invasive hemodynamic monitoring in HF patients. As in previous HF studies, sex- and weight-related differences in the hemodynamic response to diuresis were observed, and unique response-to-treatment clusters were suggested, which may be used to later understand the variability in the response to different treatments.

## Figures and Tables

**Table 1:** Demographic and baseline hemodynamic characteristics of the patients included in the study (*n* = 29). BMI, body mass index; HbA1c, Hemoglobin A1c; SPO_2_, blood oxygen saturation; HFrEF, heart failure with reduced ejection fraction (≤40%); HFpEF, heart failure with preserved ejection fraction (>40%); CABG, coronary artery bypass graft; ACE, angiotensin-converting enzyme; ARBs, angiotensin receptor blockers.

Characteristic (Mean ± SD)	
Age (Years)	75.5 ± 9.7
Sex (Male/Female)	17/12
BMI (kg/m^2^)	27.0 ± 4.5
Outpatient clinic visits per month	2.6 ± 1.7
Creatinine on the day of recruitment (μmol/L)	163.6 ± 112.7
Baseline hemodynamic parameters (mean ± SD)	
Pulse rate (beats/min)	76.2 ± 8.5
Systolic blood pressure (mmHg)	130.0 ± 30.7
Diastolic blood pressure (mmHg)	71.5 ± 18.3
Mean arterial pressure (mmHg)	91.1 ± 20.3
Pulse pressure (mmHg)	58.4 ± 24.0
Stroke volume (mL)	89.8 ± 7.0
Cardiac output (L/min)	6.8 ± 0.8
Cardiac index (L/min/m^2^)	3.9 ± 0.6
Systemic vascular resistance (dynes/sec/cm^−5^)	1080 ± 250
Smoking status N (%)	
Current	5 (17.2)
Past	12 (41.4)
Never	12 (41.4)
Diabetes status N (%)	
HbA1c (%, mean ± SD)	6.8 ± 1.4
Not diabetic	9 (31.0)
Prediabetic	4 (13.8)
Diabetic	16 (55.2)
**Heart failure type N (%)**	
HFrEF	15 (53.3)
HFpEF	14 (46.7)
**Medical history N (%)**	
Ischemic heart disease	21 (72.4)
Coronary artery bypass graft (CABG)	5 (17.2)
Hypertension	22 (75.9)
Dyslipidemia	23 (79.3)
**Medication use N (%)**	
Furosemide	29 (100)
The total daily dose of oral furosemide (mg, mean ± SD)	83.0 ± 32.9
β blockers	20 (68.9)
ACE inhibitors	4 (13.7)
ARBs	10 (34.4)
Aldosterone antagonists	13 (44.8)
Sacubitril/Valsartan	6 (20.6)
Digoxin	3 (10.3)
Metolazone	4 (13.7)

**Table 2:** Baseline characteristics between clusters (Ratios or mean ± SD). SVR—systemic vascular resistance; BMI—body mass index; SpO_2_—blood oxygen saturation; ns—not significant; HFrEF—heart failure with reduced ejection fraction; HFpEF—heart failure with preserved ejection fraction; T2DM—type 2 diabetes mellitus; VAS—visual analog scale.

	Cluster 1	Cluster 2	Cluster 3	*p*-Value	Post Hoc (*p* < 0.05)
Characteristic					
Age (Years)	74.3 ± 12.3	73.6 ± 8.7	76.8 ± 9.6	ns	
Sex (M/F)	5/1	1/6	11/5	0.02	
BMI (Kg/m^2^)	25.5 ± 5.5	28.3 ± 6.1	27.0 ± 3.3	ns	
CHF type (HFrEF/HFpEF)	4/2	3/4	8/8	ns	
Hypertension (yes/no)	5/1	4/3	13/3	ns	
Chronic kidney disease (yes/no)	5/1	2/5	12/2	0.058	
Type 2 diabetes (no/pre/T2DM)	2/2/2	1/1/5	6/1/9	ns	
HbA1c (%)	5.9 ± 0.5	7.6 ± 2.1	6.8 ± 0.9	0.085	
Creatinine (µmol/L)	274.8 ± 182.6	95.9 ± 22.4	151.6 ± 71.6	0.009	(1,2), (1,3)
Baseline parameters					
Heart rate (beats/min)	80.4 ± 8.2	69.5 ± 7.7	77.6 ± 7.8	0.038	
Systolic blood pressure (mmHg)	141.4 ± 32.9	135.8 ± 26.4	123.1 ± 31.7	ns	
Diastolic blood pressure (mmHg)	70.3 ± 26.2	64.1 ± 10.9	75.2 ± 17.6	ns	
Mean arterial pressure (mmHg)	94.1 ± 28.2	88.0 ± 15.3	91.2 ± 20.1	ns	
Pulse pressure (mmHg)	71.1 ± 10.9	71.7 ± 18.6	47.8 ± 25.1	0.025	
Stroke volume (mL)	93.7 ± 9.3	88.1 ± 7.9	89.1 ± 5.5	ns	
Cardiac output (L/min)	7.5 ± 0.8	6.1 ± 0.6	6.9 ± 0.6	0.002	(1,2), (2,3)
Cardiac index (L/min/m^2^)	4.3 ± 0.9	3.6 ± 0.5	3.8 ± 0.5	ns	
SVR (dynes/sec/cm^−5^)	1015 ± 284	1180 ± 307	1061 ± 213	ns	
Change after treatment					
VAS change (cm)	−0.1 ± 0.2	−0.5 ± 0.6	−0.5 ± 1.3	ns	
Weight loss (%)	−1.3 ± 2.3	−1.3 ± 1.4	−1.1 ± 1.6	ns	

## Data Availability

The data underlying this article will be shared on reasonable request with the corresponding author.
